# Applying theory to practice in a large research center: creating and implementing tools for building convergence capacity in individuals and teams

**DOI:** 10.3389/fpsyg.2025.1639826

**Published:** 2025-12-16

**Authors:** Kimberly Bourne, Maude Cuchiara, Alison Deviney, Daniel Laxman, Christine Ogilvie Hendren

**Affiliations:** 1 Department of Plant and Microbial Biology, North Carolina State University, Raleigh, NC, United States; 2 Science and Technology for Phosphorus Sustainability (STEPS) Center, Raleigh, NC, United States; 3 Department of Materials Science and Engineering, North Carolina State University, Raleigh, NC, United States; 4 University Office of Evaluation and Educational Effectiveness, Arizona State University, Tempe, AZ, United States; 5 Research Institute for Environment, Energy, and Economics, Appalachian State University, Boone, NC, United States; 6 Office of Research and Innovation, Appalachian State University, Boone, NC, United States

**Keywords:** convergence research, team building, integration, wicked problems, team science, interdisciplinary, transdisciplinary, and collaborative framework

## Abstract

The continued expansion of team science underscores the urgent need for shared, actionable models that can be validated to support effective interdisciplinary collaboration in scientific teams. This paper presents a practice-grounded framework for building convergence capacity in large, multi-institutional research environments, drawing on insights from the Science of Team Science (SciTS) and Integration and Implementation Sciences (I2S). Using the U.S. NSF-funded Science and Technologies for Phosphorus Sustainability (STEPS) Center as a case study, we explore how integration expertise can be strategically embedded within leadership structures to design and implement evidence-informed toolkits and conceptual models that scaffold convergence practices. These approaches operationalize convergence through three interdependent priority focal points —scaffolding effective teamwork and group integration capacity, cultivation and management of boundary objects, and investment in the integrative capacity and professional growth of the individuals that make up the teams—each supported by specific tools, facilitation strategies, and team development approaches that align with established theories of collaboration and integration. By translating convergence theory into replicable, field-tested practices, this study contributes to the development of team science models that are adaptable to diverse research contexts and challenges. We share how one example of a large research center incorporated literature- and practice-based convergence approaches into the organizational structure and research efforts from the onset of problem formulation and team formation, and we highlight how integration practitioners serve as catalysts—structuring processes, facilitating shared meaning-making, and fostering collective metacognitive awareness essential to interdisciplinary problem solving. Our reflections and observations in this manuscript emphasize the critical role that deliberate, resourced, theory-driven intervention can play in building the conditions for sustainable collaboration and effective knowledge co-creation.

## 1 Introduction

### 1.1 The case for building convergence research capacity

Convergence is a research, development, and innovation approach to address complex multifaceted societal challenges (Gajary et al., 2024) by bringing together the knowledge, methods, and expertise of different disciplines in service of problem-inspired research (see NSF, NASEM). This approach is of particular relevance to addressing the many complex problems society currently faces, such as climate change and food insecurity, in which there are networks of related systems and issues that demand simultaneous attention. Managing these complex problems, some of which are referred to as “wicked problems” (Rittel and Webber, 1973; Crowley and Head, 2017), depends upon the blending of knowledge, language, methods, and expertise across varied vantage points and skill sets. Wicked problems are characterized by a number of challenging attributes that preclude straightforward “solutions,” including the absence of a stopping rule and the inability to test by trial and error given the interconnected complex systems they exist within. In addressing such problems, particularly when there is a pressing near term need for resolution, it becomes increasingly important to develop and employ ways to enable rapid, effective integration of broad contributions toward shared solutions (Sundstrom et al., 2023). A responsible and generative approach toward building a body of knowledge about how we, as a research community, can contribute to meeting the challenges of our time must include transparent, explicit sharing of the context-specific applications of convergent research approaches as we develop, implement, and evaluate our emerging methods for this complex research. This manuscript describes the methods applied in one Center as a case study, based in evidence and literature and adapted through practice to the needs of a specific team and grand challenge problem.

While definitional consensus is elusive in this as in other combinatory, emergent fields, the authors of this paper operate with the shared understanding that convergence moves beyond transdisciplinarity by breaking down both disciplinary *and* other epistemic and sectoral boundaries to create new integrative scientific fields while including non-academic partners as critical contributors (Sundstrom et al., 2023). Doing so requires a reflexive approach to research in which knowledge is co-created or transformed—both contextually and iteratively—through cross-disciplinary and cross-sectoral interactions and collaborations (Roco, 2016; Sundstrom et al., 2023; Gajary et al., 2024).

### 1.2 Brief overview of convergence research

Thompson et al. (2023) note the importance of context to defining convergence and the dependence on perceived outcomes (e.g., addressing large-scale social issues, developing new technology, or increasing research productivity). Indeed, the concept of convergence itself has evolved from comparatively simple disciplinary models such as the economic principle describing the confluence of two inversely proportional curves (Barro and Sala-i-Martin, 1992) to the conceptual sociotechnical framework for addressing grand societal challenges by integrating disciplines introduced by Roco et al. (2013). Convergence research is now embraced by multiple sectors from policy to medicine to socio-ecological systems (e.g., Wagner, 2020; Sundstrom et al., 2023; Devine et al., 2025), and a growing body of literature supports convergence practices to address wicked problems through frameworks and empirical studies (see, for example: Angeler et al., 2020; Frechtling et al., 2021; Devine et al., 2025). Yet, as Sick and Bröring (2022) note in their review of the convergence landscape in technology and innovation management, the field of convergence research struggles with consistency in both theory and application. This underscores the utility of making evidence-based approaches explicit and transparent, as a pathway to shared understanding of how teams can scaffold and conduct convergence research.

Therefore, the effort to move beyond disciplinary collaboration toward true knowledge co-creation has in itself inspired research focused not only on actual outcomes, but on how they are achieved through adjacent fields such as the Science of Team Science (SciTS; Peek et al., 2020), and Integration and Implementation Sciences (I2S; Misra et al., 2024). These emerging fields are helping to develop, study, and refine the methods and tools for intentionally and effectively managing collaborations and interactions that drive forward the convergence process (Bammer, 2003; Fiore, 2008; Stokols et al., 2008a). Often, intentional approaches structured by the leadership in planning an effort, wherein an organization applies a particular framework to accelerate convergence outcomes, are rooted in principles derived from SciTS and I2S, providing structured guidance across teams toward a common goal.

Convergence requires significant effort to create the appropriate conditions under which it is possible. However, these efforts are not always formally established or defined in a leadership-scaffolded approach; sometimes successful convergence research outcomes may have occurred organically. Therefore, a critical aspect of the work to understand and encourage the factors that make successful integration and co-creation of knowledge possible is identifying, and then emulating the behaviors and conditions that have led to convergent outcomes. For example, in reviewing affective vs. cognitive research, Zhukov et al. (2024) demonstrate that it is the multidisciplinarity of citing fields, not the multidisciplinarity of the authorship, that best predicts impact—a pattern they describe as ‘downstream convergence.” In another study Petersen et al. (2021) describe that the pressure to converge can lead large science teams to practice “convergence shortcutting” by relying fully on external interventions of multidisciplinary or boundary-spanning individuals rather than true disciplinary integration. These studies indicate the benefit of intentionally recognizing and leveraging boundary spanning interests and expertise into tools and practices adopted within the team that support convergence research.

As the mixing zone of theory and practice continues to evolve, an academic “estuary” is created wherein the knowledge and research of I2S, SciTS, and other fields are being translated into applied methods to generate value by enabling more *intentional, effective*, and *faster* convergence (Lotrecchiano and Misra, 2020; Misra et al., 2024; Love and Dickmann, 2025). Like an estuary full of biodiverse organisms that interact and strengthen ecosystems, the intersection between the scholars and practitioners of collaboration and team-based discovery creates opportunities for applying novel approaches to team interactions in an iterative, rapidly adapting system. The possibility for such adaptation within this rich estuarine environment also encourages teams to study themselves in the process of the practice, to drive individual- and systems-level improvement in their capacity to blend epistemologies and approaches to address pressing societal challenges. Borner et al. (2010) provide a framework for understanding the who, what, when, where, and how of addressing challenges across these multiple levels of collaboration. The potential for generalizable insight from this combination of experimentation and application of evidence-based practices is ever more promising as an appreciation for, and widespread investment in, convergence research grows.

### 1.3 Integration expertise

As the need to address complex issues becomes increasingly pressing and teams grow in size and diversity, spontaneous or ad hoc attempts at research integration and implementation are no longer an adequate or responsible approach (Lélé and Norgaard, 2005; O'Rourke and Crowley, 2013; Roco and Bainbridge, 2013; Sundstrom et al., 2023). Though organic emergence of convergence research may be possible, it doesn't make sense to leave its achievement to chance when success could be aided via intentional application of emerging evidence-based methods. Small et al. (2021) illustrate multiple case studies where embedding a “Reflexive Monitor” to facilitate the collaboration dynamics of multifaceted groups supported the co-creation of solutions for complex agricultural problems. Drawing upon the previously described estuary of collaboration theory and practice, such expertise can help position large multidisciplinary teams for successful outcomes when addressing wicked problems, particularly when cultivated at multiple levels of the organization, from individuals to teams as a whole (Borner et al., 2010).

Integration experts—defined as those with knowledge of or experience in convergence practices or a related field such as SciTS and I2S—are tasked with the intellectual labor of surfacing and synthesizing connectivity across the many moving parts of an organization. While these individuals may exhibit the boundary spanning capabilities typically exhibited in polymathic convergence as described by Petersen et al. (2021), their purpose is to facilitate the growth of convergence capacity within multidisciplinary teams. This work includes bridging sub-project teams, disciplines, methodological languages, institutions, sectors, and other less apparent divides that may present a barrier to collective knowledge advancement, so that goals are advanced in a more efficient manner (Borner et al., 2010). The need for this class of professionals has been explored by scholars in a number of fields (e.g., Hoffmann et al., 2022; Cravens et al., 2022; Hendren and Ku, 2019).

The intentional inclusion of an integration expert at the outset of project development provides the instructional guidance needed to structure convergent practices within an organization's operation. Integration experts also identify, develop, and facilitate the implementation of convergence tools, methods, and processes within a team. These experts act as the “arrows,” connecting system components to steer the flow of knowledge throughout an organization, and provide a link between different nodes to translate knowledge into action (Hendren and Ku, 2019, p. 363). Like an athletic coach, integration experts plan, organize, and lead integration activities and tool deployment, enabling the team as a whole and its members as individuals to develop their own metacognitive awareness and understanding of convergence processes. Similar to the members of a sports team, each player may already be an expert in how to play their own position, but to succeed together, they must also know the rules of the game and how their position interacts with others. Furthermore, team members must develop the metacognitive habits and facilitative skills to provide collective agency over effective integration processes (Salazar et al., 2012; O'Rourke and Crowley, 2013; Misra et al., 2024). While integration experts cannot provide omniscient awareness or facilitate all knowledge co-creation, they can and do provide scaffolding to enable individuals to build convergence capacities at levels appropriate to their roles and functions within the team. This in turn leads to more adaptive and adoptable solutions to the complex societal challenges convergence research seeks to address.

Bammer et al. (2020) note that expertise specific to research integration and implementation is required to successfully bring together diverse perspectives when tackling complex problems. This ability to facilitate the development of shared language and understanding across different disciplinary and experiential views in order to create functional solution spaces is strongly supported as critical to addressing complex problems (Austin et al., 2008; Stühlinger et al., 2019). Thus, identifying and applying research integration and implementation expertise can provide a solid foundation for teamwork and knowledge co-creation, a sense of purpose for those involved, and productive cross-communication between the different factions needed to spur innovative outcomes.

### 1.4 Applying theory to practice in a large research center

While literature often illustrates theoretical benefits of convergence tools, few case studies exist that provide evidence of these tools in practice, especially in large distributed multidisciplinary teams. The Science and Technologies for Phosphorus Sustainability Center (STEPS) is a U.S. National Science Foundation-funded Science and Technology Center formed around the complex problem of sustainable phosphorus management. This case study is being shared transparently at an intentional midway point in the life of the project. In the spirit of enabling a collaborative, community-wide look at how various expressions of convergence research are functioning in a practical sense, we offer this open representation of our approach and the results as we have understood them so far. The authors want to note that some of the impacts reported here include qualitative evaluation data and others represent the observations of our integration team and Center participants, as the full set of results of our meta-studies and Center evaluations will not be publication ready for some time; evaluation data is clearly distinguished in the text. We share here a transparent case study as it is currently in progress, with a community of researchers who may be able to use this case study real-time in their own attempts toward convergence research. From its inception, STEPS was rooted in principles and theory emerging from I2S and SciTS to harness the value of an estuary community, put theory into practice, and assess and adjust those practices as needed. Thus, STEPS serves dual mutually reinforcing purposes: to address the wicked problem of phosphorus sustainability while simultaneously studying and evaluating the process and methods of convergence in a large research center.

Phosphorus is considered a wicked problem because it is essential to all life on Earth and critical to sustained food production, yet its extensive use has led to soil and surface water degradation from agricultural runoff and wastewater discharge, causing eutrophication from excessive nutrient loading resulting in harmful algal blooms, fish kills, and impaired recreational and drinking water systems (Ashley et al., 2011). STEPS seeks to address many aspects of this complex problem in parallel by combining expertise from eleven different institutions and dozens of disciplines. To accomplish this, the STEPS Center was designed with a robust convergence framework in place. From the outset a named and dedicated role of Integration Director was included within the Center Co-PIs and in the leadership team. This role is held by an integration expert, a team science practitioner who rooted the Center's integrative framework in evidence-based practices.

The Center benefits from a team of convergence practitioners mentored by the Integration Director and invested in by Center leadership. The Integration Director brings extensive Team Science and Integration and Implementation Science experience and developed the convergence framework that is the foundation for the Center's convergence practices. The practitioners lead the study and application of integration methods within STEPS to build convergence capacity across the team's various disciplines and geographically dispersed membership. Through these efforts and under the guidance of the Integration Director, the practitioners refine and adapt integration methods and gain extensive integration experience. Here the co-authors present core components of the STEPS framework that can be adapted to other contexts, organized by the three categories stated above.

This manuscript seeks to address a critical gap in the current literature wherein practical applications of theory are rarely reported in detail. The objectives of this methods paper are two-fold. Grounded in the STEPS experience, we first provide guidance for designing convergence capacity into organizations across other complex problem spaces by presenting a selection of specific tools and practices that can support an integrated approach to convergence research. Second, because organizations evolve and grow over time, we highlight challenges encountered in implementing these tools and practices and how flexibility may be maintained in the scaffolding to ensure continued engagement and effective outcomes over time.

## 2 Methods and approaches

**Figure 1:**
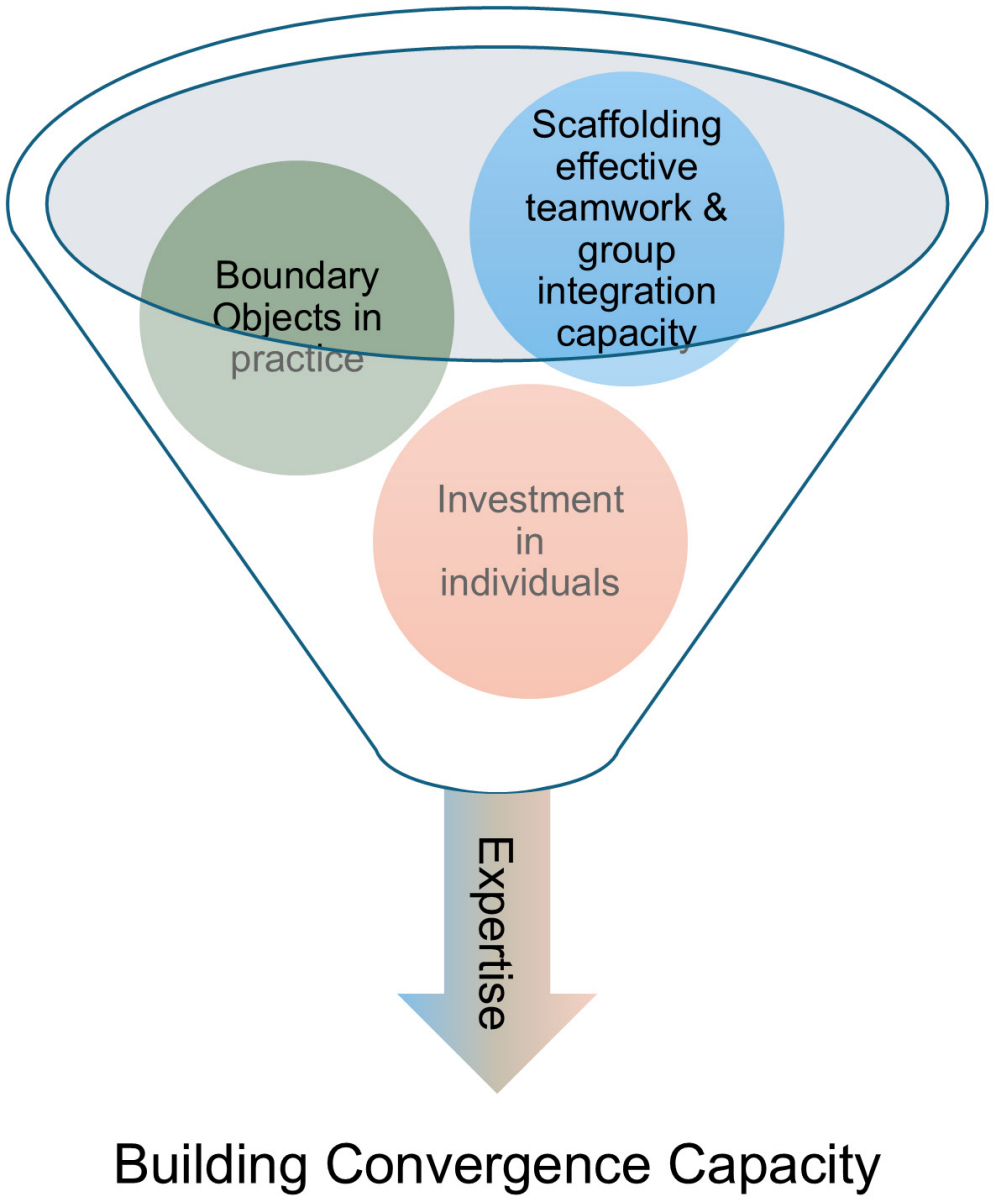
Visual representation of the process for building convergence capacity in a large research center.

**Figure 2:**
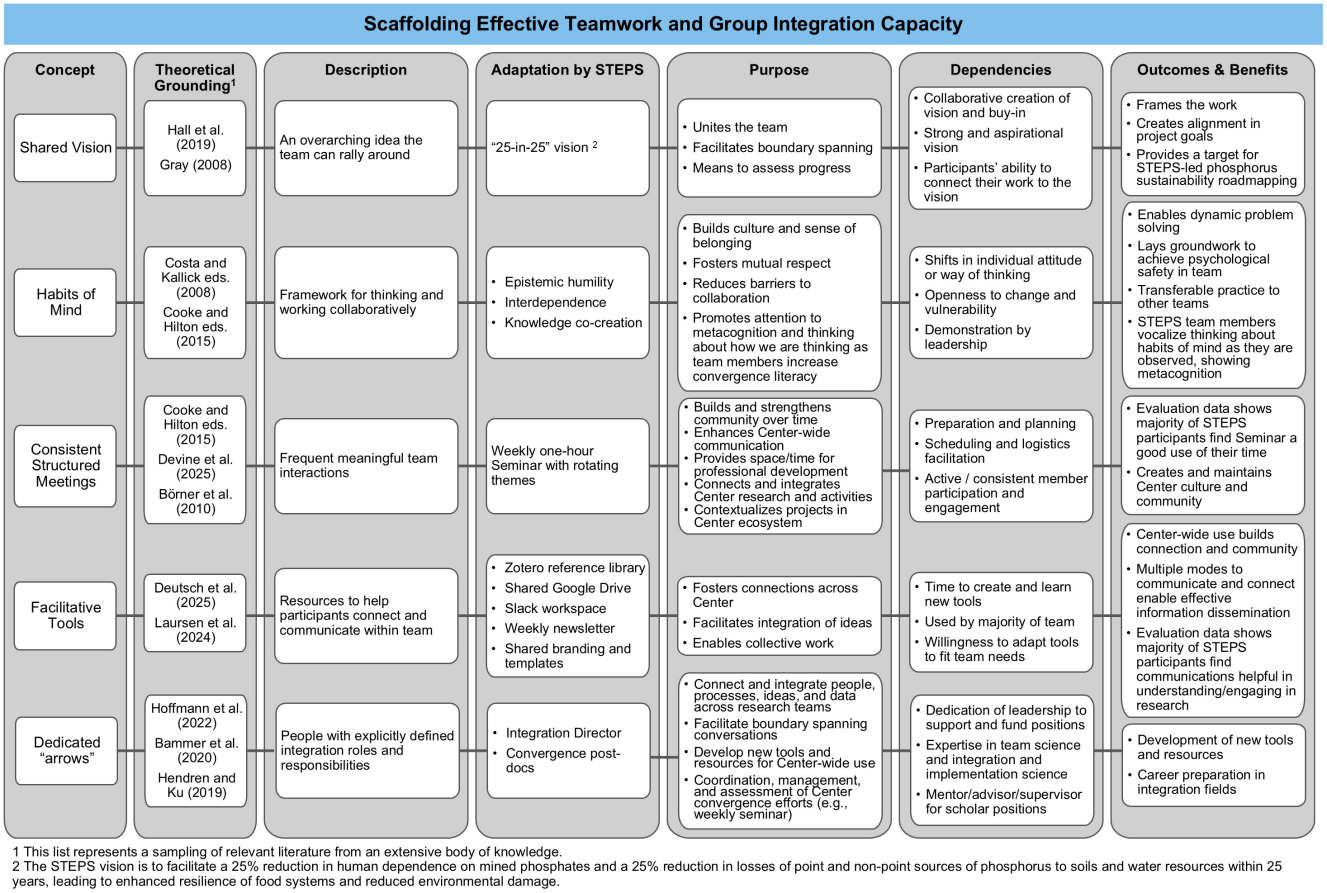
Description of tools and practices for building team cohesion. The first column lists the concept used by the STEPS Center while the second and third columns provide the theoretical grounding (i.e., the literature) from which the Center identified and adapted these concepts and a short description of the concepts. The fourth and fifth columns describe how each concept was implemented within STEPS and its intended purpose. The sixth column outlines the “dependencies” (i.e., what was needed for the concept to be successful). The last column offers the outcomes and benefits of each concept as seen or expected in STEPS.

**Figure 3:**
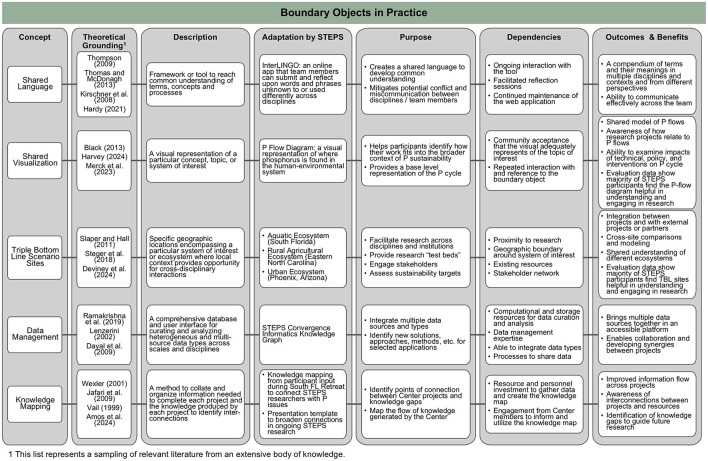
Description of tools and practices for using boundary objects. The first column lists the concept used by the STEPS Center while the second and third columns provide the theoretical grounding (i.e., the literature) from which the Center identified and adapted these concepts and a short description of the concepts. The fourth and fifth columns describe how each concept was implemented within STEPS and its intended purpose. The sixth column outlines the “dependencies” (i.e., what was needed for the concept to be successful). The last column offers the outcomes and benefits of each concept as seen or expected in STEPS.

**Figure 4:**
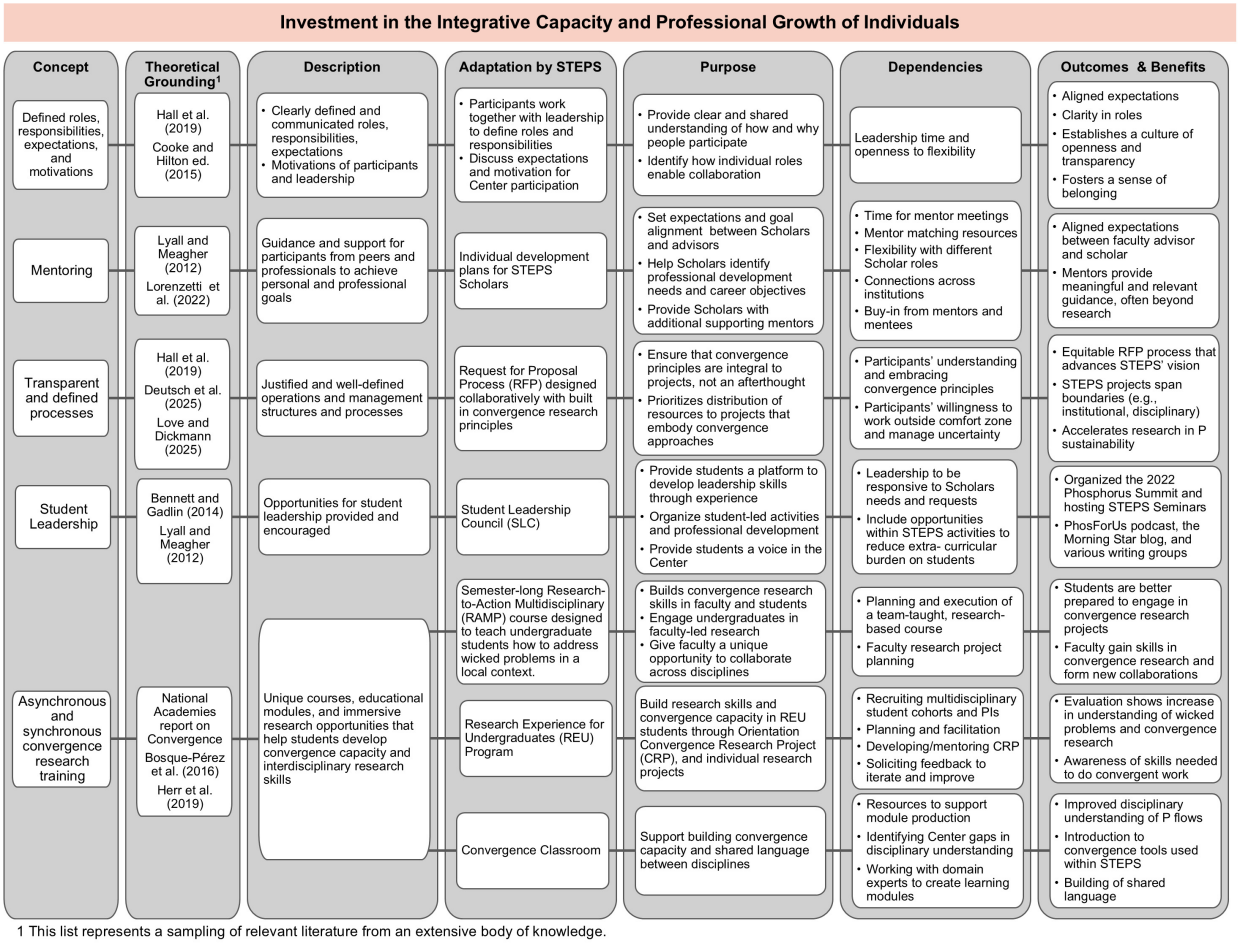
Description of tools and practices used to invest in integrative capacity within the individuals that make up the teams. The first column lists the concept used by the STEPS Center while the second and third columns provide the theoretical grounding (i.e., the literature) from which the Center identified and adapted these concepts and a short description of the concepts. The fourth and fifth columns describe how each concept was implemented within STEPS and its intended purpose. The sixth column outlines the “dependencies” (i.e., what was needed for the concept to be successful). The last column offers the outcomes and benefits of each concept as seen or expected in STEPS.

As noted above, there are numerous tools, practices, and frameworks designed to support convergence research. In this section, we describe the designation of integration experts that will deploy the tools and methods used to help translate the theory of convergence into practice by identifying a set of core components for scaffolding a team's convergence approach. Using STEPS as an example, we divide these practices into three categories that contribute to a comprehensive approach to yield team success: (1) investment in team cohesion, (2) boundary objects in practice, and (3) investment in people (as individuals and as teams; Figure 1). To make this framework more practical for implementation, the practices are illustrated in Figures 2–4, correlating to these three categories. The methods employed are rooted in and guided by convergence-supportive literature and shared here as a case study for how these approaches have been adapted to shape and guide a real research team.

### 2.1 Designating the integration expert(s)

Core to implementation of the tools and approaches for enabling this boundary spanning work is the need for qualified individuals to be named, empowered, and explicitly tasked in the team with carrying out the integrative work. In the context of the convergence approach shared here, the work of integration experts includes but also goes beyond serving as team science experts and facilitators, with the contributions of the dedicated integrator hinging on the ability and bandwidth to manage the bridging of knowledge as well as fluency in at least one of the fields engaging in the research mission that guides the team. Demonstrating and further shaping the variety of ways that integration expertise can be woven into a team has been a core commitment of the experiment that the STEPS Center team has undertaken with its convergence effort, including a continual examination of how trainees are brought into and empowered in integrative work.

At this moment in the emergence of I2S expertise, the field is still developing as a discipline. We see concepts and methods, shared language and literature, communities of practice and conference gatherings, and the beginnings of being able to measure contributions. What we do not yet see are the associated recognized degrees, departments, career pathways or journals that provide a straightforward way of developing and recruiting integrative expertise. Gabriele Bammer describes a disciplinary emergence trajectory through an analogy to the discipline of statistics, which provides a helpful example showing how the contribution and methods come first, and these drive the establishment of structured pathways to advance and practice the work (Bammer, 2017). Like statistics, not everyone who uses the methods and insights from the field in their work is themselves a dedicated statistician.

In the case of our STEPS case study, the individual in the role of Integration Director mentioned above was trained as an environmental engineer who then managed a large interdisciplinary research center. Curious about what methods may exist to optimize this integration process and elevate the priority of connective work, they slowly became more entrenched in Science of Team Science communities than they were in their home discipline, collaborating with other integrators internationally and establishing a community of practice for those in similar roles. Many integrators at the time of this publication have a similar story, with some claiming “Integration and Implementation Sciences (I2S)” as their primary discipline in grant proposals that have received funding.

### 2.2 Investment in scaffolding effective teamwork and group integration capacity

STEPS invests in approaches that create the conditions and shared experiences that enable team members, as a group, to collectively increase their appreciation for, and adeptness at, blending their expertise in pursuit of a shared knowledge production goal. Figure 2 describes these approaches, which are designed to create an environment that supports open exchange of ideas, normalizes the safety of sharing, and provides mechanisms to reflect, adapt, and change as a group. Figure 2 includes an overview of specific concepts (e.g., shared vision, facilitative tools) that STEPS embedded in the Center, the theoretical grounding of each concept, a STEPS example, dependencies needed for success, and outcomes and/or benefits resulting from the concept's use (Figures 3, 4 share a similar structure). Members of STEPS are guided by a shared vision developed early in the proposal development process; this vision guides the Center's research and collaborations and is repeated often. To enhance capacity for collaboration, STEPS also onboarded three named habits of mind to enhance individual heightened awareness and the key roles they play in convergence research: epistemic humility, interdependence, and co-creation. All team members were encouraged to use these terms in day-to-day communication and embrace them in practice during Center meetings and interactions. Additionally, team meetings provided an opportunity for integration experts and Center leaders to model this behavior, as people onboarded the meanings of these terms as tools to nudge convergent mindsets. Regular meetings structured for open exchange and reflection, facilitative tools, and dedicated integrator roles further provide the scaffolding necessary for Center members to engage in productive collaboration.

### 2.3 Boundary objects

A boundary object is an artifact that exists at the intersection of disciplinary or social worlds, creating a common ground needed to facilitate communication between differing perspectives (Stoytcheva, 2013), without necessarily requiring agreement regarding the object's definition or purpose (Leigh Star, 2010). Boundary objects can be physical entities (e.g., a visual, model, knowledge collection, or place) or conceptual (a term or idea). While there are different schools of thought regarding how individuals learn to integrate different concepts into new meaning, some theorists hold that this occurs by blending existing concepts (e.g., “problem-solving”) rather than a constructivist approach of increasing complexity by building on what is already known (Mansilla, 2017). Boundary objects support this conceptual blending approach by and across individuals by providing a shared focus. Thus, identifying what may serve as a boundary object depends heavily on the context of the research issue being explored, and the disciplinary breadth or distribution of the team involved. Concepts, models, visuals, methods, tools, datasets, or physical locations that embody a wicked problem of interest can be considered as long as they are specific enough to garner general agreement regarding their purpose, but ambiguous enough to embrace multiple disciplinary perspectives.

Caccamo et al. (2023) explain in their review of boundary object literature that it is the continuous process of disciplinary knowledge boundary spanning across different levels that leads to knowledge integration. Over time, teams consistently engage with and increasingly understand these linking concepts resulting in continuous knowledge exchange. As “intellectual watering holes,” boundary objects pull together the different threads of awareness to allow teams to speak the same language and integrate disparate data sets. They also improve understanding of individuals' relevance to one another within the context of a shared challenge (Lenzerini, 2002; Mattor et al., 2014; Merck et al., 2023). Regardless of whether a boundary object is a physical location, a visual schematic, or an abstract concept, the interactions with it give concrete form to the shared contribution across disciplines and scales. Thus, as collaboration across knowledge bases in a team progresses, the shared tool of a boundary object becomes a platform for new practices and ways of thinking to emerge, enabling the knowledge integration and co-creation that is critical to success in convergence research.

In the STEPS case study, multiple boundary objects were deployed to support cross-disciplinary integration and problem-solving for communication, concept visualization, contextualization, data management, and knowledge mapping. These concepts are described in Figure 3. Tools include a shared language app, a phosphorus (P) flow diagram, Triple Bottom Line Scenarios Sites (TBL Sites)–a concept unique to the STEPS Center–and a Convergence Informatics user interface. The inspiration for developing these tools was the idea of having tangible and namable structures to which the STEPS research community could relate both individually and collectively. While not functionally different from boundary objects in general, these tools were initially referred to as “convergence boundary objects,” (CBOs) to promote acceptance and use within the broader STEPS community not conversant in SciTS or I2S. To test this theoretical application, STEPS developed and piloted multiple visual and physical CBOs to help generate a community culture across disciplinary perspectives. Some of these boundary objects provide dedicated resources that target particular convergent practices (e.g., shared language building, data management), while others serve a more holistic purpose of disciplinary interaction and integration. The multifunctional nature of these boundary objects was intended to generate greater engagement within the STEPS community through their cross-disciplinary appeal (e.g., TBL Sites, P Flow Diagram).

STEPS also leverages its TBL Sites as boundary objects through annual “retreats” to engage a subset of its research community in site-specific cross-disciplinary and cross-institutional collaborations. Each retreat involves scholar (trainee) specific activities, and a full day tour of various field site visits to engage with and learn from local stakeholders, followed by a full-day workshop event to then brainstorm research opportunities related to “big ideas” generated from the previous day's tour and activities. A pre-retreat meeting and instructional activities “set the stage” for these Retreats, while follow up meetings disseminate findings and opportunities to the broader STEPS community and facilitate ideation follow-through.

### 2.4 Investing in the integrative capacity and professional growth of individuals

The adoption of effective tools and methods is paramount to creating the conditions for successful convergence research on complex problems but will be fruitless without direct investment in team members' individual growth in onboarding convergence capacity (Bennett et al., 2018; Love et al., 2021). This investment means dedicating concrete resources toward supporting researchers at all stages of their careers as they grow in their ability to engage effectively in convergence research to address complex problems. Figure 4 provides examples of how teams can effectively invest in individuals.

STEPS has implemented many tools, methods, and programs to support its faculty, staff, and scholars, as shown in Figure 4. Upon joining the Center, members of STEPS are prompted to clearly define their own roles and responsibilities through structured collaboration planning. Scholars—whose roles within the team are more transitional than faculty investigators—submit Individual Development Plans that describe how their work in the Center contributes to their professional growth. STEPS further supports their development through opportunities for additional mentoring outside of their direct supervisor and participation in the Student Leadership Council (SLC). In STEPS, undergraduates who are not part of a specific project may also build convergence research skills by participating in the Research Experience for Undergraduates Program or an integrative “Research-to-Action Multidisciplinary Projects” course offered at one of the partner institutions, Appalachian State University. In this way, STEPS includes training the future workforce in the convergence framework. STEPS has also identified postdoctoral fellows who are uniquely receptive and able to train as dedicated arrows under the guidance of the team's Integration Director. These early career researchers, having achieved disciplinary expertise through their doctoral programs, use their position as an opportunity to focus on deep integration across perspectives, often leading multidisciplinary publications related to convergence research. To orient a broader community of Center participants and potential partners to convergence concepts and to establish a base level literacy about the different disciplines or convergence concepts within the Center, STEPS developed a publicly available online repository of educational materials, the Convergence Classroom.

### 2.5 Assessment and evaluation

The field of evaluation as it relates to convergence methods is nascent and few established frameworks exist (Committee on National Statistics et al., 2021; Laursen et al., 2022). The team collaborated with professional evaluators to assess aspects of the Center. This work generated immediate, useful insights and laid the groundwork for future research on effective convergence evaluation. The University Office of Evaluation and Educational Effectiveness (UOEEE) at Arizona State University conducts an external evaluation of STEPS members' readiness for and engagement in convergence research and team science, complementing internal efforts led by STEPS researchers and leadership. Data collection includes a series of surveys sent to all Center participants, an annual strengths, opportunities, weaknesses, and threats (SWOT) analysis with scholars, and REU surveys and interviews that gather data on individual-level attitudes toward, preparedness for, and engagement in convergence research. Evaluators also developed items based on Nurius and Kemp's (2019) framework of 15 individual-level team science competencies in the categories of values, attitudes, and beliefs; habits of mind; knowledge-based competencies; and interpersonal competencies to better understand and support the development of skills critical for effective cross-disciplinary collaboration. Center leadership works closely with the evaluation team to inform the evaluation instruments. The Center also supports research focused on understanding the experiences of our graduate trainees (Jones et al., 2025) and conducts informal evaluation through reflective exercises during Center meetings and events.

## 3 Discussion

STEPS adapts a number of strategies, processes, and tools based in literature and to facilitate integration within a convergence center (Figures 2–4). Here we will discuss how this framework was applied in one context, but the principles and methods shared can be adapted to address other wicked problems.

### 3.1 Designating the integration expert(s)

For a large convergence research project, this team's assertion is that it is helpful to include an individual whose role in the team is that of an integration and implementation scientist. Their job is to bring in concepts and methods from the international fields they are active within [I2S, SciTS, the Inter- and Transdisciplinary Alliance (ITD Alliance), Association of Interdisciplinary Studies (AIS), and others] to support the shared mission of the Center, just as the materials scientists, plant biologists, and water engineers do with their expertise. Without any credentialing pathways that exist for integration expertise, teams must rely on self-identified integration experts to spearhead the development and deployment of convergence scaffolding.

As discussed throughout this paper, however, a single individual cannot and should not be the only source of integrative expertise and activity in a large center. To spread this capacity more broadly beyond themselves and build individual and team integration capacity, they need to share their expertise through practice and mentorship. A dedicated integrator contributes to the team by helping the group as a whole progress their understanding of the shared problem being undertaken, and by sharing this process and knowledge development with their colleagues and bringing trainees into the process who are interested in developing and practicing this integrative work. Together the integrative experts and trainees help the team navigate decisions about what actions to take, what questions to ask, and what connections exist or need to exist between people and epistemologies across the project. The model in the STEPS case study has been that the dedicated I2S professor, the Integration Director, invites others into the process of developing, applying, and improving these concepts. The broadened group of boundary spanning agents throughout the Center includes others in critical integration leadership roles (e.g., Director and Managing Director), trainees who have rapidly become critical to the integration operations of the Center and to advancing the integrative applications and ideas that underpin these operations (multiple post-docs), and many other engaged faculty and trainees who want to onboard integrative practices they find useful into their own subteams or other work.

### 3.2 Knowledge integration and co-creation through investment in scaffolding effective teamwork and group integration capacity

The Center intentionally introduced and embedded convergence scaffolding to support team integration in pursuit of shared research goals (Figure 2). Project teams and organizations focused on addressing complex problems can benefit from straightforward interventions that are explicitly taught and facilitated to enable the transfer of insight across epistemologies; encourage effective listening; and build teamwork, collaboration, cohesion, and trust in teams (Deutsch et al., 2025; Gilligan, 2021). To spur convergence, teams benefit from the ability to experience repeated interactions that help everyone onboard and value shared team goals and shared language (National Research Council, 2015; Devine et al., 2025). Integrative tools and integration experts, responsible for developing, facilitating, and managing their use in teams, can provide the scaffolding for these types of interactions and boundary spanning capacities (Hendren and Ku, 2019; Bammer et al., 2020; Hoffmann et al., 2022). Further, as team members become accustomed to these approaches, they can also adapt and apply them independently to build convergence capacity in their sub-teams and other groups.

External evaluation data across 3 years (2022–2025) demonstrate that the tools, resources, and approaches developed through STEPS have been widely adopted and become integral to STEPS members' research and practice. As a brief illustration, evaluation findings indicate strong growth and/or consistency in both use and perceived helpfulness of each tool for engaging in convergence research. Across years and evaluation instruments, sample sizes ranged from 24 to 41 for faculty/staff and from 30 to 48 for students and postdocs. STEPS Weekly Seminar sessions (focused on activities, facilitated sessions, and presentations related to convergence research) were attended by 88–97% (varies by year) of faculty/staff and students/postdoc evaluation survey respondents, with 81–98% rating the tools as “moderately,” “very,” or “extremely” helpful for engaging in convergence research. Group brainstorming templates were also used extensively, with 76–95% of respondents indicating use and 79–93% reporting high levels of helpfulness, particularly in the most recent year. The use of the curated shared Zotero library has grown from around 70% for all STEPS members in 2022–2023 to 77% and 91% among faculty/staff and students/postdocs, respectively, in 2024–2025. Finally, communication from STEPS leadership about activities, research, and opportunities was reported as helpful by 98% of evaluation survey respondents with near universal engagement. Together, these findings demonstrate that STEPS members have widely adopted these tools and see them as particularly helpful and impactful for engaging in convergence research in the Center.

### 3.3 Boundary objects in practice

At the outset of STEPS, boundary objects represented the ripest, highest impact opportunity to directly apply an established yet versatile concept in transdisciplinary literature within the foundational fabric of the Center. The broad adoption of this concept throughout the Center has steered much of STEPS convergence from the inception of the team. The collection of boundary objects developed by STEPS and outlined in Figure 3 have helped build a systems-level understanding of the interdependencies of phosphorus flows and related socio-environmental systems, providing anchors for interaction and allowing each researcher to understand the context of their own work in the bigger picture.

The P flow diagram, which provides a representation of phosphorus flows through nodes and arrows, is among the most widely recognized and adopted boundary objects within STEPS, with various efforts to “improve” the diagram by either adopting it to regionally specific P flows or adding additional nodes or groups (see, for example, Merck et al., 2023). Additionally, the TBL Sites provide specific bounded geographic ecosystems within which researchers from different disciplines integrate their expertise to engage with stakeholders and test innovations in real-world settings. TBL Sites provide important contextual understanding across disciplines as well as the ability to compare data and intervention outcomes across different contexts.

External evaluation data across 3 years (2022–2025) provide an illustration of how boundary objects are impacting STEPS members and research. For example, 83–100% (varies by year) of faculty/staff and student/postdoc respondents reporting use of the P-flow diagram and 75–84% benefitting from the TBL Sites. Faculty/staff and students/postdocs rated these tools as beneficial, with 88–95% of respondents rating the P-flow diagram and 82–91% rating the TBL Sites as “moderately,” “very,” or “extremely” helpful for engaging in convergence research. Use and ratings of the shared language app were collected during the 2023–2024 grant year. Use of the app was common (71% of faculty/staff and 64% of students/postdocs), and while ratings of the app by faculty and staff were somewhat lower during that time, nearly two-thirds of students and postdocs (63%) rated the tool as “moderately,” “very,” or “extremely” helpful for engaging in convergence research as it continued to be refined.

Evaluation data also highlight the impact of the TBL Site Retreats held in January 2024 (27 evaluation survey respondents) and September 2025 (33 evaluation survey respondents), with 100% of faculty/staff and student/postdoc survey respondents rating each retreat as a “good” or “very good” use of time and funding. Similarly, 92% of faculty/staff and 87% of student/postdoc respondents rated the January 2024 retreat as “very” or “extremely” helpful in engaging in convergence research, with 80% and 85%, respectively, giving the same rating for the September 2025 retreat. For example, one faculty member shared how attending the 2024 retreat helped them better place themselves and their work in the Center in regard to other STEPS members:

“My participation in the retreat helped me gain a better understanding of my own purpose in the Center. Prior to the visit, I was not sure how to make my position relevant to others across the center; however, during the retreat, I was able to feel more like an included member of the center.”

The retreats also help faculty, staff, postdocs, and students apply the tools and resources developed by STEPS to strengthen teamwork and integration skills in a practical, hands-on way, as illustrated by one scholar's comments:

“[The retreat] helped me better understand how to facilitate brainstorming sessions. It has also helped me gain more insight into potential changes to the knowledge mapping methodology.”

### 3.4 Investing in the integrative capacity and professional growth of individuals

As a convergence center, the goal of STEPS is not only to achieve convergence as it relates to phosphorus sustainability, but to also train the next generation of convergence scholars. For individuals to understand how they can contribute to shared goals in a large convergent team, they must be able to navigate the broad expanse of contributing disciplines and contexts while managing the potential risks of information overwhelm, uncertainty, and a lack of clarity on expected contributions. By focusing on individualized mentoring, roles and expectations, and communication norms that include metacognition around convergence processes and their challenges, Center leaders can effectively support the people who make up the teams undertaking convergence research. Prioritizing individuals can help defray the potentially increased stress brought about by the challenge of encountering and judiciously assimilating such a broad range of input knowledge sources, shepherding them into the process of building their own individual and team capacities for integration. Even experienced researchers need to invest in expanding their expertise to include convergence methods to help them manage uncertainty inherent in working across disciplines and on wicked problems. Trainees (defined within STEPS as “scholars,” include post-doctoral fellows, graduate students, and undergraduate students) often require additional support to operate confidently within this uncertainty as well. With convergence research becoming increasingly necessary (Committee on National Statistics et al., 2021; Gajary et al., 2024), it is essential that teams invest in scholar development in particular.

External evaluation efforts have tracked outcomes related to convergence research and team science knowledge and skills over time, examining how individuals in the Center grow in their convergence capacity. As an illustrative example, analyses of evaluation data from 2022 to 2025 indicate a consistent increase year over year in the proportion of STEPS members who self-rated their understanding and ability to apply skills as “proficient” or better on a nine-point scale with definitions. For each of the following outcomes, the proportion of STEPS members rating themselves as “proficient” or better was greater each year: understanding of convergence research (68% in 2023–2023 to 84% in 2024–2025 for faculty/staff; 46–81% for students/postdocs), understanding of team science (66–91% for faculty/staff; 61–83% for students/postdocs), applying convergence skills (70–80% for faculty/staff; 44–71% for students/postdocs), applying team science skills (70–84% for faculty/staff; 50–76% for students/postdocs).

### 3.5 Limitations, lessons learned, and next steps

Our experience highlights the value of a dedicated integration director to selecting the tools that are contextually appropriate, acting as the “arrow” to foster shared language, create and use boundary objects, teach integration capacity within peers and trainees, and facilitate and encourage collaboration (Bammer et al., 2020; Cravens et al., 2022; Deutsch et al., 2025; Gajary et al., 2024). An embedded integration leader helps teams determine when and how to pivot to different tools/methods of convergence, aided by mechanisms to solicit generative feedback through formal or informal evaluation of this scaffolding.

When the person in this role also mentors peers and trainees, they proliferate the integrative behaviors and skills to other dedicated integrators as the organization matures. Within STEPS, we have seen the professional growth of postdoctoral fellows as they developed these skills. Their roles include building expertise through leadership positions within integration communities of practice (Interdisciplinary Integration Research Careers Hub, or INTEREACH, INSciTS), organizing convergence workshops and conference sessions, and leading integration efforts across research teams and in newly funded programs outside of the Center. Thus, as the convergence literacy of the team broadens, multiple leaders can adapt and apply integration methods so that all participants have an opportunity to onboard the language and concepts that enable convergence. This has been demonstrated at multiple levels, including in smaller teams within the Center. For example, at the start of a new research project focused on biological phosphorus removal in wastewater, one team intentionally identified the poly-phosphorus granules found in phosphorus accumulating organisms as a boundary object. The work of each researcher could be tied back to these granules, providing a connective tissue between projects.

STEPS has also illustrated how convergence takes a consistent—and considerable—investment of time and therefore monetary investment. This is an important consideration given that barriers at the institutional level often exist that can present additional challenges to implementing a convergence approach, such as latent incentive structures and inadvertent competition in scenarios where individuals are seeking to collaborate (Lélé and Norgaard, 2005; Stokols et al., 2008b; Vogel et al., 2014). As previously noted, Petersen et al., 2021 describes the rise of polymathic convergence as a mode of so-called “convergence shortcutting” to circumnavigate the costs of cross-disciplinary convergence and underscores that these shortcutting efforts fail to achieve the deep integration necessary for addressing scientific grand challenges. They advocate for policy changes to require and support classic convergence. The benefits of implementing integration practices are not always immediate or obvious and depend upon dedicated investment in the communication and collaboration norms that build up sufficient trust and shared vision to navigate around such barriers in service of the motivating mission. However, these benefits of convergence are reflected in the quality of team performance, research outputs, and the long-term achievements in innovation application and adoption.

Assessment and evaluation of the structured collaboration systems adopted by a team or center are essential to the success of that system (Gajary et al., 2024; National Academies of Sciences, Engineering, and Medicine, 2025). Each individual and team has different needs for building and maintaining convergence capacity, necessitating early, consistent evaluation to provide dedicated integrators with the feedback they need to adopt or sunset convergence tools (Misra et al., 2024) Here, we share a real experimental system, a “center as a laboratory” model for building and running a convergence research center. The approach has been systematically designed based on literature rooted in I2S and SciTS, but by nature of being among an early generation of such endeavors, the ability to assess its successes against a literature-supported set of evaluation tools does not yet exist. Instead, appropriate evaluation metrics are concurrently emerging and being tested by experimental application within convergence science centers alongside proposed assessment tools. Insights gleaned from these experiments appear in the “Outcomes and Benefits” columns of Figures 2–4 and are described above. A critical part in this process is dedicated time, as a Center, to discuss the challenges and issues that are identified through evaluation. In this, the Center works together as a team to develop solutions, strategies, and best practices that are then implemented. The team seeing that leadership is responsive helps to build community and trust.

The STEPS Center continues to evolve based on feedback from our participants, our evaluation strategy, and the development and assessment of convergence methods. Early evidence from this transparently shared case study suggests that the convergence and integration framework and approaches developed and applied within this organization have led to innovation and built individual and team convergence capacity. As the life of this research team approaches seven years and the data generated in the official Center funding period reaches 5 years, natural future inquiries will include a quantitative assessment of our convergence framework guided by methods described in Devine et al. (2025), Huffman et al. (2025), and Nurius and Kemp (2019) and comparisons of the convergence scaffolding in STEPS to that of other centers and developing metrics to assess the effectiveness of the methods, processes, and tools meant to foster convergence.

## 4 Conclusion

The intentional cultivation of integration expertise, literacy and practice across multiple organizational levels can provide important support for teams charged with successfully addressing complex or wicked problems within large, convergence research teams. The STEPS Center develops, applies, and adapts evidence-based convergence research approaches to address the wicked problem of phosphorus sustainability, providing insights for other teams. Preliminary evaluation demonstrates that despite conceptual and scientific challenges, the inclusion of such intentional support yields multiple benefits including collaborative, interdisciplinary research outcomes. Methods necessary for convergence will not be uniform across teams and will vary based on the needs of individual groups; this fact underscores the importance of openly sharing case study applications of convergence research approaches within their unique contexts to provide examples for other teams. The framework and the STEPS Center case study presented here offers such a transparent example alongside the literature bases that have informed the approach, providing a practical starting point for implementation in other systems. It is our hope that the intentional application of convergence research strategies as outlined in this guide may help other stakeholders of convergence research navigate the challenges of complex research teams and achieve desired outcomes.

## Data Availability

The original contributions presented in the study are included in the article/supplementary material, further inquiries can be directed to the corresponding authors.
